# funcExplorer: a tool for fast data-driven functional characterisation of high-throughput expression data

**DOI:** 10.1186/s12864-018-5176-x

**Published:** 2018-11-14

**Authors:** Liis Kolberg, Ivan Kuzmin, Priit Adler, Jaak Vilo, Hedi Peterson

**Affiliations:** 1 Institute of Computer Science, University of Tartu, Juhan Liivi 2, Tartu, Estonia; 2 Quretec Ltd, Ülikooli 6a, Tartu, Estonia

**Keywords:** funcExplorer, Gene expression, RNA-seq, Microarray, Protoarray, Hierarchical clustering, Functional enrichment analysis, Global visualisation, Data-driven

## Abstract

**Background:**

A widely applied approach to extract knowledge from high-throughput genomic data is clustering of gene expression profiles followed by functional enrichment analysis. This type of analysis, when done manually, is highly subjective and has limited reproducibility. Moreover, this pipeline can be very time-consuming and resource-demanding as enrichment analysis is done for tens to hundreds of clusters at a time. Thus, the task often needs programming skills to form a pipeline of different software tools or R packages to enable an automated approach. Furthermore, visualising the results can be challenging.

**Results:**

We developed a web tool, funcExplorer, which automatically combines hierarchical clustering and enrichment analysis to detect functionally related gene clusters. The functional characterisation is achieved using structured knowledge from data sources such as Gene Ontology, KEGG and Reactome pathways, Human Protein Atlas, and Human Phenotype Ontology. funcExplorer includes various measures for finding biologically meaningful clusters, provides a modern graphical user interface, and has wide-ranging data export and sharing options as well as software transparency by open-source code. The results are presented in a visually compact and interactive format, enabling users to explore the biological essence of the data. We compared our results with previously published gene clusters to demonstrate that funcExplorer can perform the data characterisation equally well, but without requiring labour-intensive manual interference.

**Conclusions:**

The open-source web tool funcExplorer enables scientists with high-throughput genomic data to obtain a preliminary interactive overview of the expression patterns, gene names, and shared functionalities in their dataset in a visually pleasing format. funcExplorer is publicly available at https://biit.cs.ut.ee/funcexplorer

**Electronic supplementary material:**

The online version of this article (10.1186/s12864-018-5176-x) contains supplementary material, which is available to authorized users.

## Background

The amount of high-dimensional biological data produced by next-generation sequencing and microarrays is continuously increasing. Clustering is one of the most popular analysis approaches to get a quick overview of patterns present in data. Well-known clustering algorithms, such as the hierarchical clustering [1], self-organising maps (SOM) [2] and k-means [3] approaches are mostly applied to group together genes with similar expression profiles [4, 5]. Once the clusters are known, the prior knowledge of Gene Ontology (GO) [6], and other functional databases, such as KEGG and Reactome [7, 8], is utilised to interpret how and why the genes in a cluster are related to each other and to interpret these results in the context of previous knowledge. For this purpose, functional enrichment analysis is used to identify which of the known annotations are over- or under-represented in a cluster [9]. The output of such analysis is a list of enrichment *p*-values for each biological annotation. Finally, the results are gathered and represented visually to convey the obtained information at a glance.

This pipeline is often conducted manually. For example, the hierarchical clustering of breast tumor expression profiles revealed a large cluster of distant metastasis-free survival-associated genes with known immunological functions identified by GO enrichment analysis [10]. A similar approach was used by Yang et al. to show that diurnal genes in the prefrontal cortex of mice fall into eight temporal categories with distinct functional attributes [11]. The gene expression patterns of 200 lymph node-negative breast cancer patients were hierarchically clustered and the identified co-regulated gene clusters were shown to be related to the biological process of proliferation, steroid hormone receptor expression, as well as B-cell and T-cell infiltration by Schmidt et al. [12].

However, the common analysis pipeline presented above has several pitfalls. For example, most of the standard clustering methods depend on preselected parameters like the number of clusters in k-means or the size and architecture of the SOM map. The height to cut the dendrogram, a tree-like arrangement where every node denotes a gene cluster [13], in hierarchical clustering needs to be set to extract the clusters. The latter is often evaluated by visual observation or by trial and error. A key limitation of these approaches is that they rely heavily on the researcher’s prior knowledge or assumptions and judgment, and thus are biased [14–17]. This undermines the reproducibility of scientific discoveries. In addition, as clustering often results in multiple gene groups and there exists a bulk of different annotation sources to compare against, the number of computations required is potentially very large [17]. Therefore, manual selection of interesting clusters followed by functional enrichment analysis on each of these clusters is very time-consuming and often impractical. Due to the lack of complete pipelines from clustering to visualisation, several tools need to be applied successively to obtain the desired analysis and this may be an obstacle to a biologist without programming skills. Furthermore, we can assume that researchers perform this pipeline many times with various parameters and choose to present the results of their preference without acknowledging all the preceding attempts.

Multiple tools have been developed to integrate clustering and functional enrichment analysis, but all of them have key limitations (Additional file 1: Figure S1). For example, desktop applications such as Expander, AMEN and HCE [18–20] require additional installations and expensive local computations and disk space. An R package CLEAN [21] combines biological knowledge with the results of a cluster analysis, but it requires additional tools for interactive visualisation and provides a limited selection of biological data sources. An R package clusterProfiler [22] automates the process of biological classification and the enrichment analysis of gene clusters, but does not cluster the dataset itself and requires programming skills. Clustergrammer [23] offers web-based interactive heatmap visualisation together with clustering and functional enrichment analysis opportunities. However, the enrichment is calculated to provide only one cluster and data source at a time. In addition, the tool facilitates cherry-picking of clusters as there is no automatic approach for cluster selection. Krushevskaya et al. introduced an enrichment-driven pruning method for hierarchical clustering of gene expression data together with a visualisation interface called VisHiC [24]. The idea was to detect clusters from a dendrogram based on significant enrichments, revealing the interesting clusters in the clustering tree while hiding less relevant parts from the output.

Here we present funcExplorer, a much improved and updated implementation of VisHiC. The present tool is a complete rewrite of its predecessor. The analysis pipeline, methods and measures are thoroughly updated in funcExplorer. The whole interface of the web application is modernised to offer a better user experience and more opportunities to explore data. The funcExplorer analysis incorporates automatic hierarchical clustering and functional enrichment analysis to find data-driven gene clusters from data. We have utilised the g:Cocoa tool in the widely used and regularly updated g:Profiler toolset [25] to deliver up-to-date information from numerous public databases such as Gene Ontology, KEGG, Reactome, Transfac [26] and Human Protein Atlas [27]. The results have been combined into a compact heatmap dendrogram that highlights the most biologically relevant clusters and hides poorly annotated expression profiles. This visualisation is presented in a user-friendly graphical interface to give a fast comprehensive overview of the functional essence of the data and provide a means to interactively explore the data in detail without the need to download any additional software. The user can adjust the visualisation by modifying the parameters for clustering, thresholds for enrichment *p*-values and different annotation categories of interest. A short URL that redirects to obtained results can be generated to share them with colleagues or provide them as a reference in a publication for other researchers to explore and interpret.

With the increasing availability of data and advancements in technology and visualisation, making sense of biological data without bioinformatic skills is problematic. Our goal is to enable biologists to perform a basic analysis pipeline without the help of bioinformaticians or the needs for high-end programming skills and get results that can be readily interpreted. At the same time, the tool is also beneficial to bioinformaticians to obtain a quick overview of data before conducting a thorough analysis. The tool can be accessed at https://biit.cs.ut.ee/funcexplorer and the source code is freely available from https://gl.cs.ut.ee/biit/funcexplorer_public.

## Implementation

**Fig. 1:**
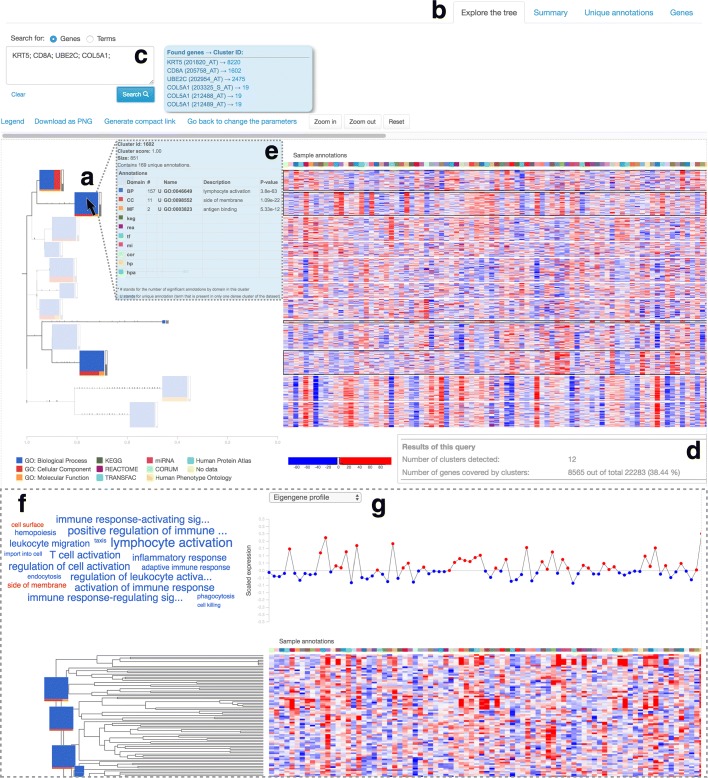
Global overview of the Humoral dataset GSE11121 in the user interface of funcExplorer.**a** The main output shows a compact dendrogram with a heatmap aside. Clusters, indicated as rectangles, are size-scaled and color-coded according to significant functions from enrichment analysis. **b** Additional information in the form of tables is shown in tabs ‘Summary’, ‘Unique annotations’ and ‘Genes’. **c** Search field allows searching for interesting genes or functions. The results will be reported next to the field and are highlighted in the main view by dimming unrelated clusters. **d** Bottom of the page shows a report of output (number of clusters, gene coverage) and selected parameters (not shown in the figure). Clicking on a specific cluster or cluster-link redirects the user to a single cluster view. **e** Hovering over a cluster shows a tooltip with the most relevant information. **f** Wordcloud in the single cluster view shows functional topics; **g** Expression profiles characterise the behavior of the cluster across samples. The results of this figure are available for more detailed exploration at https://biit.cs.ut.ee/funcexplorer/link/22ebb

We have revived the original ideas of VisHiC by developing funcExplorer to obtain fast data-driven functional characteristics of high-throughput gene expression data without requiring extensive code writing from the user. funcExplorer automatically carries out the first steps in experimental data analysis and thereby saves scientists valuable time and resources. This is accomplished through an easy-to-use web interface that performs automatic functional clustering analysis resulting in interactive visualisations, summary statistics and functionalities that facilitate the analysis of the underlying biology of data (see Fig. 1).

Revisions to the methods and measures include:

- A new clustering algorithm: Hybrid hierarchical clustering [28].
- Better integration with g:Profiler and thereby an increased number of available functional categories and gene identifiers.
- Revised initial strategy measures for detecting clusters.
- Inclusion of new measures with a clear description of expected outcomes: F1 measure and first annotation.
- Exclusion of size-weighted annotation score.
- New data upload system developed with enhanced possibilities such as RNA-seq normalisation, missing value imputation, background selection for enrichment analysis, sample annotations and many more.
- Modern GUI provided with extensive improvements in functionality and content.
- More thorough cluster descriptions achieved with eigengene profiles and functional topic wordclouds.
- Increased maintainability due to the new modularised implementation realised using Linux containers via Docker.
- Software transparency achieved by open-source code.

In the following sections we present the capabilities of funcExplorer in further detail.

### Overview of funcExplorer workflow

The input of funcExplorer can be any high-throughput gene/protein activity data. For example, it supports expression data from RNA-seq or microarray experiments, or autoantibody activities from ProtoArray. The input format is a standard data matrix where gene/protein identifiers fill the rows, samples fill the columns and corresponding activity levels fill the cells. The analysis is not restricted to expression values only. In principle, any numerical activity measure of genes across a number of conditions is admissible. The accepted file formats include custom delimited files as well as SOFT formatted files from the Gene Expression Omnibus (GEO) repository [29]. A successful upload automatically launches the analysis pipeline in the back end.

A personal URL of form https://biit.cs.ut.ee/funcexplorer/user/83dfcffcba4067a43dc53025dbb1620a, where *83dfcffcba4067a43dc53025dbb1620a* denotes the user or dataset-specific random hexadecimal key, is returned to the user after being uploaded. The specific results are accessible from the URL after the calculations are finished. This allows everyone with the link to go to the page and explore the results for themselves or change the visual parameters and analyse the dataset further. This can be a useful supplement to a research publication that is based on ideas that emerged from the data in funcExplorer. We keep the results for 365 days by default, but on request we are able to keep the data for an extended period of time. For example, if the dataset link accompanies a scientific publication, we are willing to keep the dataset indefinitely.

It is recommended, but not mandatory, to provide an e-mail address when uploading data. If an e-mail is given, the URL will not be disclosed on the web page for security reasons. The user will receive an e-mail notification with the relevant URL when the results are ready. Moreover, providing an e-mail enables the user to access all previously uploaded experiments from a single page later on. At the same time, the URL system does not impose another password to be remembered.

In addition, it is possible to directly explore the results by selecting publicly available datasets obtained from the ArrayExpress database [30], which can be identified by their accession numbers (for example, E-TABM-53, E-GEOD-10691). These have been preloaded and preprocessed for the tool MEM [31], and shared to funcExplorer. Altogether there are already more than 230 experimental datasets from 15 different species available for exploration. The dimensions of the available datasets vary from 5 to 685 samples and include over 54,000 transcript identifiers. In this way we also facilitate and encourage the reuse of public scientific data to increase the value and interpretability of already available data. (See Additional file 2 for a more detailed description of input data.)

**Fig. 2:**
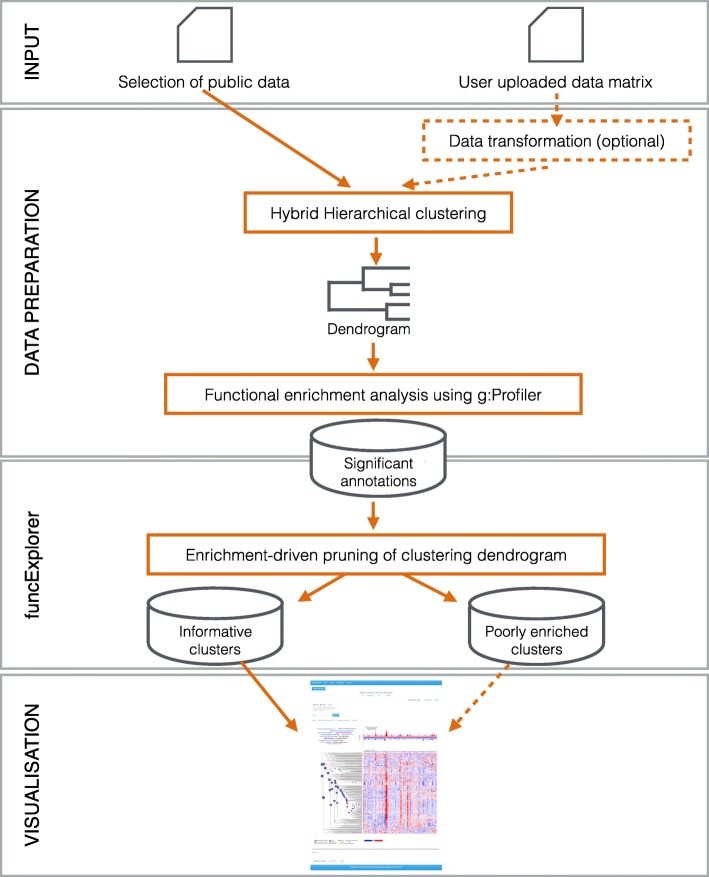
General pipeline of funcExplorer. Rectangles represent processes and cylinders represent data collections. The arrows indicate the direction of data flow while dashed arrows denote the optional path. The input dataset is gene expression data in a standard tab-separated form. Data preparation and analysis are carried out automatically and lead to a user-friendly interactive visualisation in the web browser, available for self-discovery

At its core, funcExplorer performs a series of common analysis steps on the input data. The basic pipeline comprises four main parts (Fig. 2): (1) selecting or uploading the input data; (2) preparing the dataset for analysis by hierarchical clustering and functional enrichment analysis; (3) detecting the informative and uninformative clusters based on the functional annotations and (4) visualising the results in a compressed manner with interactive components.

### Hybrid hierarchical clustering and functional enrichment analysis

The amount of data from genome-scale measurements of gene expression levels introduces several computational challenges including the duration of the hierarchical clustering and functional enrichment analysis in funcExplorer. The calculation of pairwise distances from a high-dimensional data such as gene expression data is a known computational bottleneck in hierarchical clustering.

An approximate agglomerative hierarchical clustering of a gene expression matrix is performed using the Hybrid Hierarchical Clustering algorithm [28]. The hybrid approach combines the advantages of the partitioning and agglomerative hierarchical clustering algorithms. The idea of hybrid approach is to partition the data using the k-means clustering algorithm [3] in order to avoid the full distance matrix computation. Average-linkage hierarchical clustering is thereupon applied to the partitions and to the objects within them. The linkage determines the distance between clusters as a mean value of the pairwise distances between genes in the clusters. As a result we obtain a dendrogram where leaf nodes represent single genes, all inner nodes consist of two smaller clusters and the root node contains all the genes in the dataset. The hybrid approach was shown to produce hierarchical trees similar to those of exact hierarchical algorithms [28]. Furthermore, it was demonstrated that the method is much faster, reasonably accurate and applicable to much larger datasets such as those encountered in functional genomics. In funcExplorer, we employ Pearson correlation distance as a similarity measure in hybrid hierarchical clustering as in the case of gene expression data we are often more interested in the shape of the profiles than the difference in magnitudes.

The hierarchical clustering is followed by mapping the discovered gene groups (i.e. all the nodes in the dendrogram) onto functional information based on a bulk of existing annotation sources. In funcExplorer, the functional enrichment analysis is carried out using g:Profiler software [25]. g:Profiler, like many other gene set enrichment tools, applies a cumulative hypergeometric test to evaluate whether a particular functionally defined group of genes is represented more than expected by chance within a gene cluster. The functional enrichment analysis could be performed on all possible gene clusters, i.e all nodes in a dendrogram. However, in order to reduce the computation time, we exclude very small (<5 genes) and large (>1000 genes) clusters from the enrichment analysis. A function is considered to show significant over-representation in a gene cluster if it has a *p-value*≤0.05 after multiple comparison correction. The user can choose to further narrow the range of cluster sizes and tested annotation sources or set a more stringent *p*-value threshold for annotations. (See Additional file 2 for details about statistical testing and the correction methods discussed here.)

Depending on the source availability of the organism, we profile gene clusters for GO functions [6], pathways from Reactome [8] and KEGG [7], regulatory motifs from Transfac [26] and microRNA target sites from miRBase [32], CORUM protein complexes [33], protein expression data from Human Protein Atlas (HPA) [27], and Human Phenotype Ontology (HPO) [34]. These are all the functional sources provided by the g:Profiler tool. The sources are regularly updated in g:Profiler and thereupon also in funcExplorer.

As an example, the aforementioned preprocessing of a data matrix with 22,283 rows and 75 columns takes approximately one hour to complete in funcExplorer. This includes two-way clustering of the data and annotating the clusters in parallel using g:Profiler. In order to handle the computation time in an user-friendly manner, we resolve the tasks of data preparation and visualisation separately. This means that the uploaded data are first prepared in the background and the user then receives an e-mail notification, if the email has been provided, when the dataset is ready for interactive exploration in funcExplorer.

### Detecting biologically relevant clusters

We do not want to restrict the user with a list of single clusters, but want instead to emphasise that the whole dataset is interrelated. For this purpose, we apply hierarchical clustering and harness the nested structure of the resulting dendrogram in the process of cluster detection. Our goal is to determine the collection of biologically relevant clusters of the gene expression dataset that reveals the functional essence of the data. Therefore, as a better alternative to the standard strategy of cutting the dendrogram at a fixed level of similarity across the entire dendrogram, we employ an approach that searches for the optimal cutting point from among different levels of the tree based on the functionality of gene groups measured through functional enrichment analysis. The enrichment-driven method was initially developed in the VisHiC web tool [24].

The various cutting heights are detected by maximising a predefined enrichment score within every dendrogram branch. That is, after the hybrid hierarchical clustering and functional enrichment analysis, we assign an enrichment analysis-based score to every node in the dendrogram. The scores proposed in VisHiC included a size-weighted annotation score, which summarises the negative logarithms of enrichment *p*-values, and a best annotation score, which assigns the best log *p*-value to each cluster.

**Fig. 3:**
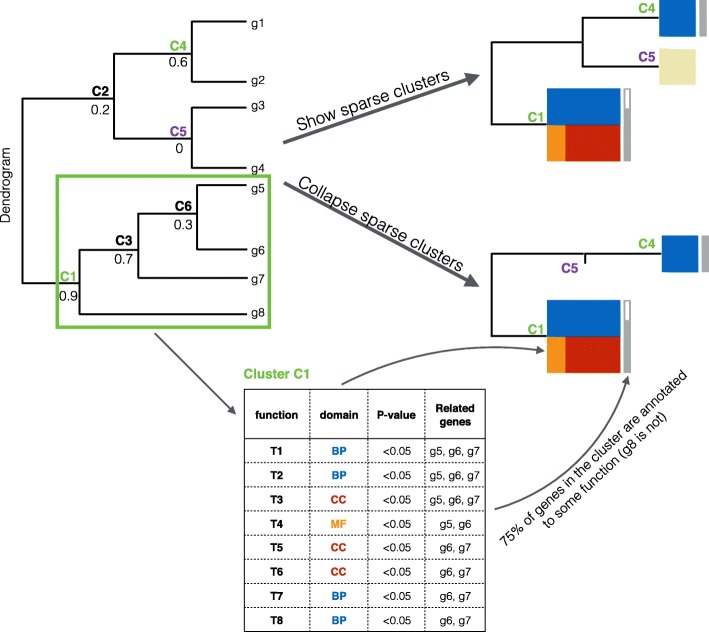
Compressing the dendrogram. The output of funcExplorer is compressed by showing only informative clusters (C1 (highlighted with green rectangle), C4) with colorful size-scaled rectangles. The colors denote the domains of significantly enriched functions (*p-value*≤0.05) in the cluster and are proportional to the number of annotations. The gray bar represents the proportion of annotated genes in the cluster. The enrichment scores used in the cluster detection algorithm are shown for every node below the cluster ID. If the ‘Show sparse clusters’ is selected, the clusters with no significant enrichment are shown with beige rectangles (C5), otherwise they are completely collapsed from the output

We start detecting the clusters from the node that has the highest enrichment score and compress it as a cluster (cluster C1 in Fig. 3). We exclude the child and parent nodes of this cluster from the further searches (clusters C3 and C6 in Fig. 3). Then we search for highest score from the remaining nodes (cluster C4 in Fig. 3) and again exclude the corresponding child and parent nodes (cluster C2 in Fig. 3). We continue these steps in the decreasing order of the scores until there is no branch left with a non-zero enrichment score. Then we traverse the dendrogram recursively starting from the root, compressing all the remaining nodes that have poor or no functional enrichment (cluster C5 in Fig. 3). Corresponding profiles are hidden from the main result of funcExplorer. However, users can include them in the output under the option “Display sparse clusters”. A full description of the algorithm is given in the Additional file 2. In funcExplorer we reimplemented the algorithm in Python, reconsidered the enrichment scores and devised new measures. See Additional file 2 for full details on calculation of enrichment scores in funcExplorer.

**Fig. 4:**
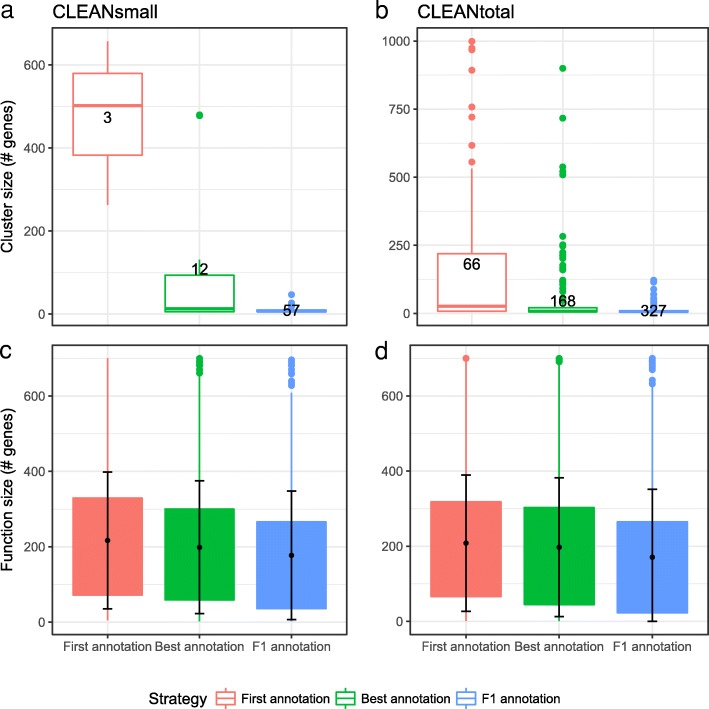
Properties of funcExplorer clusters in the example of CLEANsmall (**left**) and CLEANtotal (**right**) data analysis. **a,b** Cluster sizes after applying three cutting strategies to function-size-filtered (<700 genes) data. The number shows the total number of informative clusters detected. **c,d** The distribution of significant function sizes in the clusters with error bars (one standard deviation from average)

We added a simple **first annotation cutting strategy**. This method is based on the clustering hierarchy and will deliver the largest clusters representing a broad overview of functional features in the data (Fig. 4). The enrichment-based clusters are detected from the pre-annotated dendrogram by level order traversal of the tree starting from the root and stopping in the first node of every branch that has any significant enrichment. We define this node as a relevant and informative cluster and continue the search in neighboring branches.

The **best annotation strategy** from VisHiC remains the same in funcExplorer. In order to find clusters characterised by the “best” annotation, we evaluate the enrichment of a cluster based on a maximum of negative log *p*-value scores calculated over all significant annotation terms of the cluster. As the analysis in the following section shows, the resulting clusters are medium-sized (Fig. 4).

While reviewing the strategies, we observed that due to the discrete nature of hypergeometric distribution used for enrichment analysis, the size of a gene cluster impacts the absolute enrichment *p*-values. This makes it difficult to directly compare clusters of various sizes. In this setting we achieve the maximum possible negative log *p*-value score of a cluster only if the cluster coincides with a functional class. Corresponding behavior of theoretical best log *p*-values for various cluster sizes across different functional class sizes is shown in the Additional file 1: Figure S2. Therefore, a small gene cluster cannot achieve as high of a score as a much larger cluster due to the higher statistical power of the latter. Consequently, the best annotation strategy score prefers larger clusters related to more general functional terms.

Based on these observations, we implemented an approach to measuring enrichment in a cluster that adapted the F1 score [35] for each cluster-function combination as a stand-in for the effect size measure. In general, the F1 score is used to assess the quality of the overall clustering result, and not only for individual clusters. Here we adapt this measure to our setting and employ it as an enrichment measure by calculating the F1 scores of a cluster with respect to each of its significantly enriched functions. This gives us an intuitive way to measure the enrichment of a cluster within a fixed range of values (from 0 to 1). An analogous F1 score has previously been applied to each cluster-pathway combination by Uygun et al. to assess whether cluster membership can serve to predict pathway membership [36].

Similar to the best annotation strategy, for every cluster in a dendrogram we assign a score which is the maximum value of F1 scores over all significant annotations in the cluster. In funcExplorer we call this approach the **F1 strategy**. We are interested in functional coherence of clusters and, intuitively, this approach looks for clusters that are “complete”, i.e. have most genes from a functional category in a subtree and a large proportion of a subtree belongs to that category. We achieve the perfect F1 score with the value 1, if a cluster coincides with a functional annotation class. Our analysis revealed that this method results in small clusters characterised by specific functions (Fig. 4). This was the expected result as a majority of classes in the annotation databases are rather small and therefore the occurrence of complete overlap is more probable in smaller clusters.

### funcExplorer web interface

Once the set of relevant clusters from the input data is detected, funcExplorer visualises them in a dendrogram plot together with a heatmap. Parts of the dendrogram that have poor or no functional enrichments are hidden from the output. The detected clusters are highlighted with color-coded rectangles that carry the information of the significant functions in the cluster. The proportion of annotated genes is shown next to each cluster. This way we present a lot of information that is compressed into a compact view demonstrated in a simplified form in Fig. 3. Color-coding is also used in annotation tracks above the heatmap to describe the samples. All the visualisation components on the page are highly interactive to enable instant information retrieval from the output. In addition, the output can be easily imported to a PNG image.

The web interface provides several new options for the user to choose from. For example, the user-selected functional categories enable the user to narrow the analysis to only molecular functions from GO or choose to combine the knowledge from Reactome pathways and GO. The option to limit the size of functional categories subjected to enrichment analysis is also available. This might be beneficial when searching for highly specific annotation terms.

We have added the possibility to recursively zoom-in on the data up to the gene level through clickable elements on the dendrogram. funcExplorer has increased search that enhance the exploration of data. In addition to searching genes, we also enable users to search for functions of interest. The search results are clearly highlighted in the funcExplorer output.

To simplify cluster characterisation and comparison, a summary of gene expression profiles for each cluster is shown with an *eigengene profile*. This profile is defined as the first principal component of a given cluster. We implemented the eigengene calculation based on the WGCNA [37] R package. Additional wordclouds in the single cluster views help to get a fast overview of the functional topics of a cluster.

The results of funcExplorer analysis can be explored in all modern web browsers. The GUI of funcExplorer predominantly uses D3.js [38] as its visualisation component on the client’s side. It handles most of the rendering of the data within the web browser using a combination of standard HTML “div” and “canvas” elements. The data are sent to the web browser as JSON objects.

## Results

### Case study: Tissue-specific RNA-seq data from the GTEx project

To demonstrate the utility of funcExplorer, we uploaded RNA-seq data from 53 human tissue samples from the Genotype-Tissue Expression (GTEx) project [39, 40]. We used the dataset that contains median Transcripts per Million (TPM) values by tissue. The data matrix included 42,548 genes which includes pseudogenes and long non-coding RNAs (lncRNAs). The dataset is available for browsing from the funcExplorer page https://biit.cs.ut.ee/funcexplorer/user/3b5a789fc221a3c08a4742caccf8aeaa.

The **best annotation strategy** with a significance threshold *p-value*≤10^−8^ detected 62 clusters from the GTEx data which cover only 29.33% of the genes in the dataset. This is an expected result as pseudogenes and lncRNAs mostly lack functional annotations, and funcExplorer employs functional enrichment analysis in the clustering process. In addition, the strict significance threshold leaves out the groups of genes with low functional signal.

**Fig. 5:**
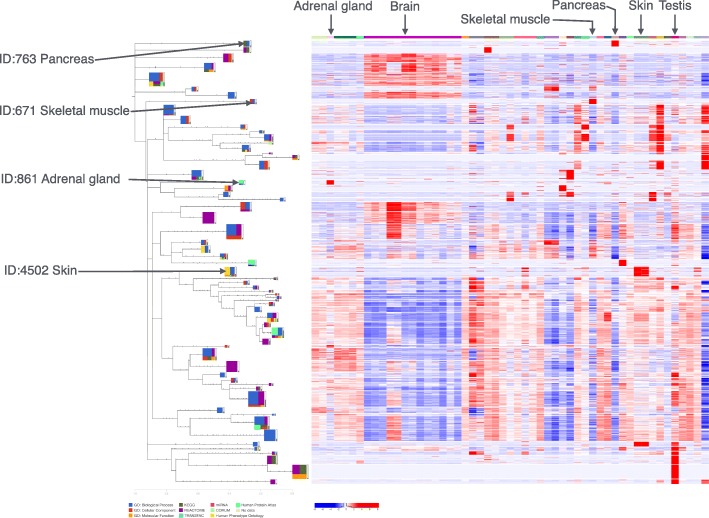
Tissue-specific clusters from GTEx data (I). Some examples of tissue-specific clusters detected by the funcExplorer **best annotation strategy** are highlighted in the figure. The results of this figure are available for more detailed exploration at https://biit.cs.ut.ee/funcexplorer/link/6afa1

**Fig. 6:**
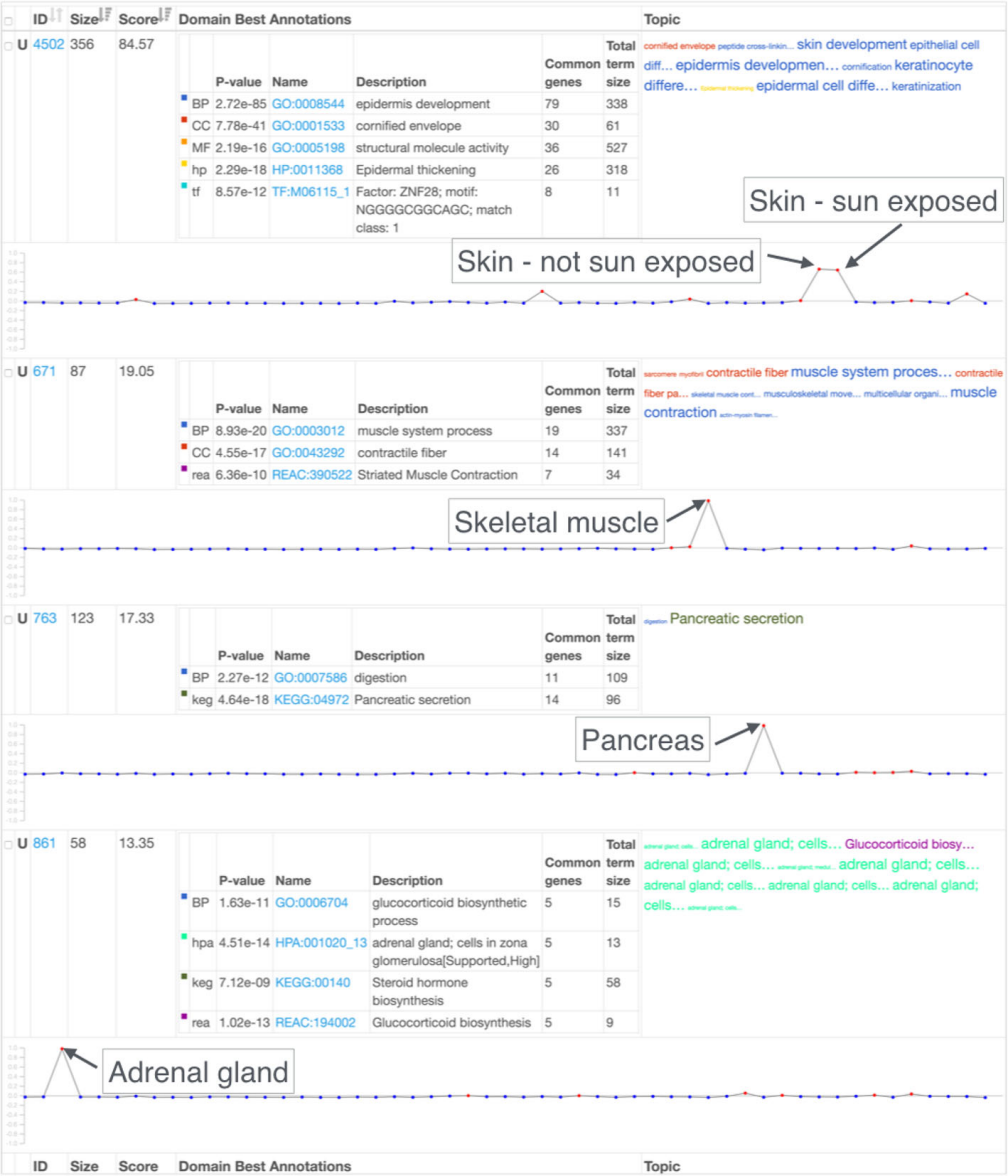
Tissue-specific clusters from GTEx data (II). A downloadable summary report of selected tissue-specific clusters detected by funcExplorer. U is an indicator of the presence of unique annotations in a cluster. The top functions of every domain are shown in the *Domain Best Annotations table*. Up to 20 top functions are represented in the topic word cloud. Eigengene profile is a representative of the expression levels of a given cluster across all the tissues. Clusters highly expressed in a specific tissue are significantly enriched with corresponding tissue-related functions. The results of this figure are available for more detailed exploration at https://biit.cs.ut.ee/funcexplorer/link/6afa1

Many of the clusters are self-explanatory and give proof-of-principle validation. We highlighted some of these in Figs. 5 and 6. For example, genes expressed only in pancreatic tissue (cluster 763) are enriched in the pancreatic secretion (KEGG:04972). Clusters where genes are highly expressed in skin (both sun-exposed and non-sun-exposed) are significantly enriched with the epidermis development process (GO:0008544). Similarly, genes expressed in skeletal muscle are functionally related to the muscle system process (GO:0003012). A cluster with a high expression in the adrenal gland is significantly enriched with adrenal gland-related functions (HPA:001010, HPA:001020).

In addition to tissue-specific clusters, we also observe that in testis there are more highly expressed genes than in other tissues. The prevalence of red color in the testis column of the heatmap in Fig. 5 indicates this. This knowledge should be taken into account in future analyses as outlier tissues might start to dominate the results. The same observation was also made by the GTEx Consortium [40].

The analysis of the GTEx data was performed without any programming or applying multiple bioinformatics tools. Just by taking the available data matrix and uploading it to the funcExplorer we got a quick bird’s-eye view of the data. This included information on how well the genes in the data were annotated in various functional databases, whether the discovered clusters were consistent with the experiment, what the leading biological processes were, and from where potential further analysis issues might emerge.

### Comparison of the three strategies provided by funcExplorer

In order to demonstrate the properties of different strategies in funcExplorer, we analysed an integrated breast cancer dataset that consists of four independent gene expression datasets with the GEO accession numbers GSE1456, GSE3494, GSE7390 and GSE11121. The same dataset was analysed as a showcase for a similar method called CLEAN [21].

We analysed two versions of the dataset: one is the preprocessed (filtered and standardised) data from the CLEAN article [21], while we assembled the other one ourselves using raw data from the GEO database. Hereinafter we refer to these two versions as the CLEANsmall and CLEANtotal dataset, respectively. The CLEANsmall dataset consists of 1422 preselected genes and 808 samples. The CLEANtotal dataset has 13,351 genes and 808 samples. Note that the CLEANsmall dataset is a subset of CLEANtotal.

To keep the analysis simple and comparable, we consider only GO annotations in this comparison. We exclude the electronic annotations as these relations are often inferred from gene expression similarity and therefore the information sources are not independent. In addition, we filtered out GO functions that are annotated to more than 700 genes as this excludes the top 5% of more general functions that characterise the dataset rather than specific clusters.

First, the datasets were analysed using the funcExplorer approach. This means that after the datasets were clustered using hybrid hierarchical clustering, all clusters ranging between 5 to 1,000 genes were extracted from the dendrogram (703 clusters altogether in CLEANsmall and 5,573 in CLEANtotal) and all these clusters were annotated with GO terms using g:Profiler with default correction method for multiple testing and all known annotated genes as a background in functional enrichment calculations (*N*=17,105, without electronic annotations). We investigated all the strategies available in funcExplorer and thus obtained three different partitions of both datasets.

The distribution of obtained cluster sizes is shown in Fig. 4a. Comparing the different strategies reveals the properties of the approaches. The **first annotation strategy** leads to large general clusters (3 altogether), while the **best annotation strategy** (12 clusters) is somewhere in between and **F1 strategy** returns many small clusters (57 clusters). Similar patterns appear also in the CLEANtotal data (Fig. 4b).

However, there is no notable difference in the distribution of the sizes of significant functions in the detected clusters across the three strategies (Fig. 4c, d). This shows that even though the clusters are selected based on only one function, the accompanying functions will not vanish from the results. For example, if a user is interested in small, specific clusters resulting from the **F1 strategy**, we will also report the general functions that appear to be significant in those clusters.

### Fixed-height cutting versus various cutoff values

Unlike the traditional fixed-height cutting, funcExplorer detects clusters from various levels of dendrogram. To show the advantage of our approach we compared the funcExplorer results with the clusters obtained from the fixed height cutting of the CLEANsmall dendrogram. We applied various cutoff values, from 0.3 to 2 with steps of 0.05 (altogether 35 cuts). After cutting we selected clusters between 5 and 1000 genes and measured their enrichment (Additional file 1: Table S1). We observed a substantial loss of information when applying fixed height cutting. For example, **F1 strategy** detected 57 significantly enriched clusters from the CLEANsmall data; however, the maximum number of annotated clusters from the fixed-cut method was 28 (cut at height 1.3; see Additional file 1: Table S1). In addition, even though the cut at height 1 (and height 1.15) extracted 35 clusters varying in size between 5 and 1000 genes, only 22 of these clusters are significantly enriched with any functional category. A similar difference also appears at other heights.

We acknowledge that in funcExplorer, we maximise for cluster enrichment which could lead to false positive results. However, at the same time, we also take into account the expression correlations through the nested structure of the dendrogram. Unlike the fixed-cut method, funcExplorer does not assume that all the clusters in the data have an equally strong correlation which enables users to reveal possible hidden clusters ensconced deep down in the tree.

**Fig. 7:**
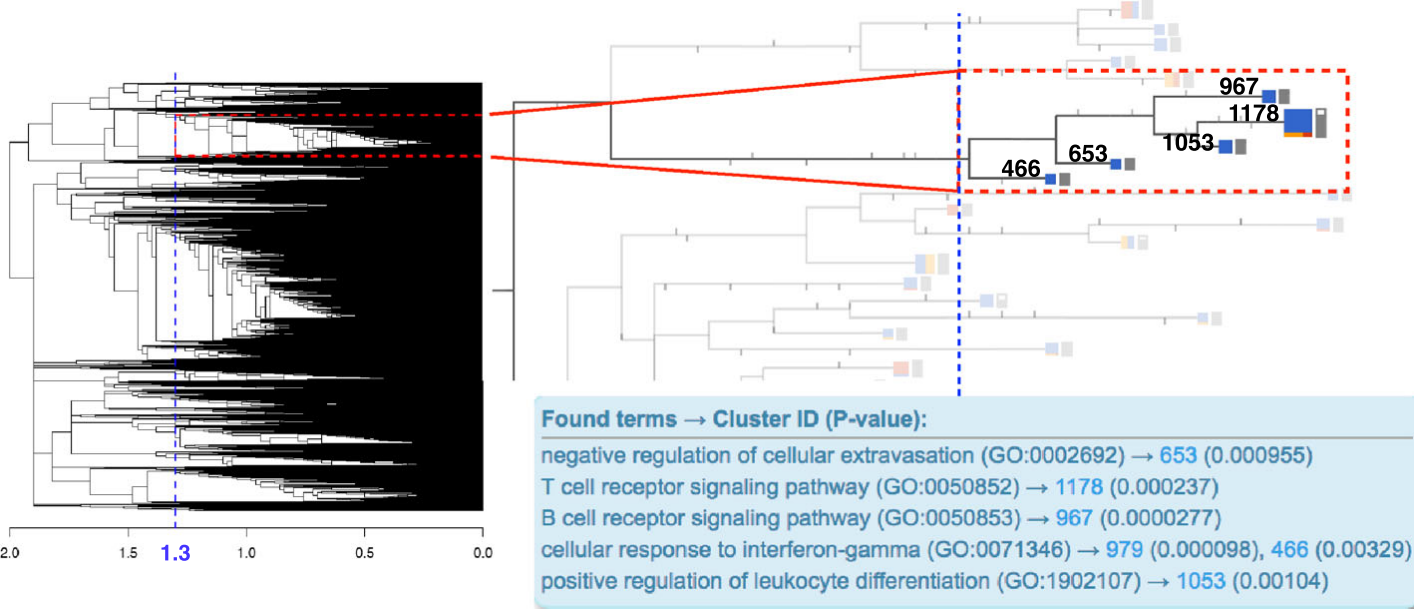
Fixed-cut at *h*=1.3 versus F1 strategy in CLEANsmall. The red rectangle on top of the dendrogram on the left highlights an example cluster obtained from the fixed-cut approach at a height of 1.3 (blue line). The F1 strategy (on the right) detected five smaller clusters from the same branch that come from various levels of tree height and cannot be detected together using one single cut. All these clusters are defined by a unique GO term which indicates a functional difference between these gene groups. For example, cluster 1178 is enriched in the T-cell receptor signaling pathway, whereas cluster 967 is enriched in the B-cell receptor signaling pathway

An example of such a situation is illustrated in Fig. 7. Here, we zoomed in on a single branch of the CLEANsmall dendrogram that was extracted as a cluster using the fixed-cut method at the height leading to the largest number of annotated clusters (*h*=1.3) (highlighted in Fig. 7 with red rectangle on the left). The top three significant GO functions enriched in this cluster are lymphocyte activation (GO:0046649), T-cell activation (GO:0042110) and leukocyte cell-cell adhesion (GO:0007159) (see Additional file 3).

While the initial immune response related cluster is split into five smaller ones with the **F1 strategy**, we still see the major functional enrichment terms (T-cell and lymphocyte activation in the cluster 1178; leukocyte cell-cell adhesion in the clusters 1178 and 1053). But in addition we can observe more specific clusters such as the clusters 967 (B-cell receptor signaling pathway; GO:0050853), 466 (cellular response to interferon-gamma; GO:0071346) and 653 (negative regulation of cellular extravasation; GO:0002692). The three clusters reveal functions which otherwise reside at the bottom of the enrichment list and are likely to be overlooked. Moreover, these clusters are characteristic to immune response subtypes, i.e. cellular immunity that involves activation of the T-cells, antibody based humoral immunity mediated by the B-cells, and a component of innate immune system as many innate immune cells are activated in response to interferon-gamma.

These observations indicate that the approach of cutting at variable heights of dendrogram branches can find clusters and highlight functions that cannot be detected using the fixed-cut method. The whole process of this comparison demonstrates how difficult it is to convey the reason of a cutting level selection. Moreover, the preceding example illustrates the benefits of hierarchical clustering to enable following the relations between the gene clusters as the five clusters in Fig. 7 reside in the same branch of the dendrogram and could be roughly described as gene clusters related to lymphocyte activation and immune response.

### Comparing funcExplorer results with previous studies

To evaluate the automated analysis of funcExplorer in its ability to produce biologically meaningful clusters, we compared the funcExplorer clusters with the results of alternative clustering methods. We used funcExplorer to reanalyse four previously published datasets that are characterised by clustering and functional annotations. These datasets cover three different organisms and illustrate a selection of typical gene clustering analysis. Thus, we have compared the funcExplorer results with the outcome from a common research approach.

In addition to the CLEANsmall dataset, we compared with three different datasets which we refer to as Humoral, Arabidopsis and Yeast. For each of them we calculated the Rand index [41] to quantify the similarity between two given clusterings. The Rand index measures how often the two genes are present or are not present in the same cluster in both of the clusterings. The similarity score exists in the range [0,1], where 1 corresponds to identical clusterings. The adjusted Rand index adjusts for the expected number of chance agreements and the value can be negative. As funcExplorer does not force genes into clusters, we also calculated a filtered version of the Rand indexes where we ignore all unclustered and scattered genes in either partitions and define the filtered Rand index only based on the intersection of clustered genes of the two partitions. The results are available in Table 1.

**Table 1:** Comparison of funcExplorer results with previous studies

Previous studies					funcExplorer											
					First				Best				F1			
Dataset	Organism	n	k	C (%)	k	C (%)	Rand(Adj.Rand)	Filt. Rand(Adj.Rand)	k	C (%)	Rand(Adj.Rand)	Filt. Rand(Adj.Rand)	k	C (%)	Rand(Adj.Rand)	Filt. Rand(Adj.Rand)
CLEANsmall [21]	H. sapiens	1,422	8	100%	3	100%	0.74(0.39)	0.74(0.39)	**7**	**93.95%**	**0.82(0.49)**	**0.81(0.50)**	33	58.09%	0.76(0.32)	0.91(0.76)
Arabidopsis [42]	A. thaliana	4,982	8	74%	7	80.08%	0.86(0.47)	0.94(0.81)	8	79.45%	0.88(0.50)	0.96(0.86)	**8**	**78.13%**	**0.88(0.50)**	**0.96(0.87)**
Humoral [12]	H. sapiens	2,579	11	46.5%	4	98.68%	0.66(0.11)	0.66(0.12)	**16**	**81.5%**	**0.72(0.16)**	**0.74(0.22)**	36	30.52%	0.55(0.10)	0.80(0.39)
Yeast [43]	S. cerevisiae	1,340	6	100%	2	100%	0.52(0.07)	0.52(0.07)	21	49.85%	0.68(0.18)	0.78(0.29)	**31**	**28.96%**	**0.55(0.11)**	**0.81(0.16)**

^*^Note: n = number of genes in the data; k = number of clusters; C (%) = gene coverage (% of genes distributed into the *k* clusters); Rand = Rand index (value between 0 and 1); Adj.Rand = Adjusted Rand index (corrected-for-chance version of the Rand index; value between -1 and 1); Filt. Rand/Adj.Rand = indexes calculated after excluding unclustered genes; the values used in the further comparisons are highlighted in **bold**; funcExplorer parameters for CLEANsmall and Humoral: *p-value*≤0.001; Arabidopsis: *p-value*≤10^−7^; Yeast: *p-value*≤0.01; no limit on term size

funcExplorer reports all the biologically meaningful clusters and does not provide any parameter to fix the number of clusters beforehand. Therefore, we can find data-driven subclusters that are clustered together in previous studies, or the other way round. We cannot achieve identical results with different number of clusters. Another way to compare the clusterings is to search the funcExplorer results for the corresponding biological functions and marker genes reported in the previous studies. This way we evaluate if funcExplorer is able to reveal previously published results.

However, it is important to emphasise that none of the compared clustering methods provide the ground truth clusters as there is rarely a gold standard indicating which genes should cluster together and which genes should be in different clusters.

#### CLEANsmall dataset

Freudenberg et al. reported 8 clusters in the CLEANsmall dataset detected by the CLEAN method [21]. The **best annotation strategy** with *p-value*≤0.001 and no upper limit to term size is the closest by detecting 7 clusters from the CLEANsmall data. The funcExplorer clusters are available at https://biit.cs.ut.ee/funcexplorer/link/c6bc4.

The Rand index for this comparison was 0.82, while the adjusted Rand index was 0.49. The filtered Rand indexes remained similar (0.81 and 0.5). Keeping in mind that the funcExplorer clusters cover 93.95% of the genes and the CLEAN method covered 100%, the results imply that the two sets of clusters are similar. The results of corresponding clusters obtained with the biological function and gene search are available in Table 2.

**Table 2:** Comparison of funcExplorer results of CLEANsmall dataset

CLEAN clusters[21]	Corresponding funcExplorer clusters	
	Cluster ID (size)	Top GO terms BP, CC, MF
1. **Nuclear part**, RNA processing (126 genes)	ID: 117 (250 genes)	Cell cycle, macromolecular complex, nucleic acid binding
2. Mitochondrion (125 genes)		
3. Mitosis, **Cell cycle** (73 genes)	ID: 63 (71 genes)	Mitotic cell cycle process, nuclear lumen, protein binding
4. DNA replication, **Cell cycle** (42 genes)		
5. **Extracellular region**, organ development, **cell adhesion** (126 genes)	ID: 5 (657 genes)	Multicellular organismal process, intrinsic component of plasma membrane, receptor activity
6. **Extracellular region**, organ development, cell adhesion (168 genes)	ID: 5 (657 genes)	Multicellular organismal process, intrinsic component of plasma membrane, receptor activity
	ID: 91 (77 genes)	Extracellular matrix organization, proteinaceous extracellular matrix
7. **Immune response**, **signal transducer activity**, **receptor activity**, **response to stress** (288 genes)	ID: 8 (170 genes)	Immune system process, plasma membrane, receptor activity
	ID: 305 (105 genes)	Immune system process, plasma membrane part, receptor activity
8. **System process**, **receptor activity**, **plasma membrane** (474 genes)	ID: 5 (657 genes)	Multicellular organismal process, intrinsic component of plasma membrane, receptor activity

^*^Note: The **best annotation strategy** with *p-value*≤0.001 and no upper limit for term size; cluster IDs ordered by the enrichment score; cluster sizes are given in the brackets; the characteristic functions of CLEAN clusters that also appear significant in the corresponding funcExplorer clusters are highlighted in **bold**; TOP functions are from GO biological process (BP), cellular component (CC) and molecular function (MF)

#### Arabidopsis dataset

Chupeau et al. performed hierarchical clustering analysis of 5,276 differentially expressed genes from transcript profiling at various time points during the preparation and culture of *Arabidopsis thaliana* protoplasts [42]. The values in the analysed data matrix are not the gene expression levels in different conditions but represent the changes in transcript levels over time. The authors detected 8 main clusters that cover 74% of the genes.

We analysed the same dataset in funcExplorer and compared the clusters (see Table 1). All of the three strategies in funcExplorer resulted in similar clusterings and these clusters were well consistent with the ones reported by Chupeau et al. [42]. In addition, funcExplorer clusters cover more genes in the data which is a proof that funcExplorer exploits the whole dataset and, instead of a favorable subset, reports all the meaningful clusters.

**Fig. 8:**
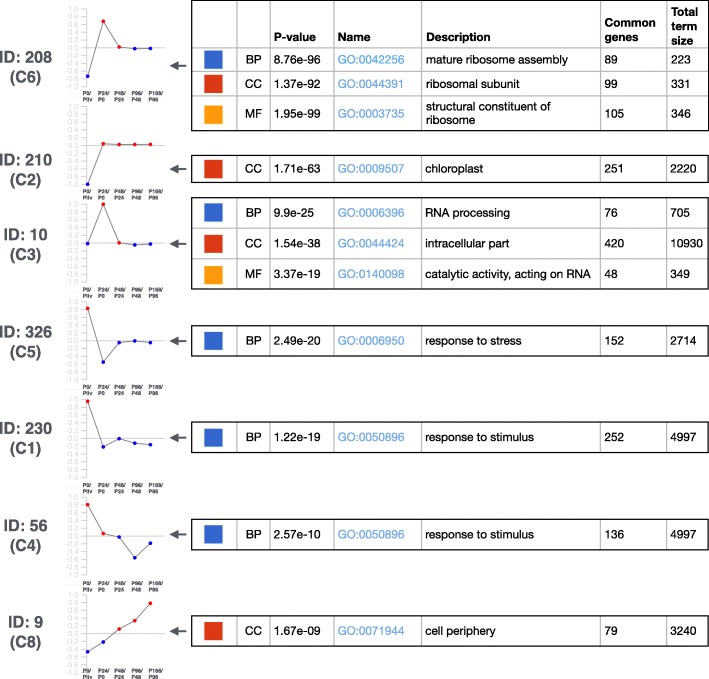
Comparison of clusters from the Arabidopsis data. The seven clusters from funcExplorer **F1 strategy** analysis that match with the clusters reported in the previous study [42]. The funcExplorer cluster IDs with the equivalent cluster name from [42] (in the brackets) are shown on the left. The eigengene profiles and significant functions that describe the clusters are consistent with the previous analysis

Searching for the characteristic functions and marker genes reported in the supplementary material of the initial analysis [42], we aimed to find the corresponding clusters from the results of the **F1 strategy** with a threshold for *p-value*≤10^−7^ and no upper limit for the term size. We were able to recover all the clusters with the exception of cluster C7. This is an expected result as the cluster C7 has been reported as a cluster with no significant enrichment in GO and funcExplorer relies on the enrichment analysis. The schematic representation of the seven clusters in Fig. 8 shows that funcExplorer is capable to achieve almost identical results with the previous study. In addition, we found the cluster 301 which is enriched in the photosynthesis (GO:0015979) and several other functions that are not significantly enriched in any of the neighboring clusters. The results of this analysis are available at https://biit.cs.ut.ee/funcexplorer/link/36e71.

#### Humoral dataset

We reanalysed a breast cancer dataset with the GEO accession no. GSE11121 first analysed by Schmidt and colleagues [12]. We refer to this dataset as *Humoral*. The dataset consists of gene expression patterns of 200 tumors of patients who were not treated by systemic therapy after surgery. One part of the analysis process was hierarchical clustering of a subset of the data (2579 preselected probe sets) to identify coregulated gene groups.

The authors defined 11 gene clusters through manual selection of branches of the dendrogram as suggested by the occurrence of cluster regions within the heatmap. However, this approach is highly subjective and another scientist could have made a slightly different choice depending on their research question. The selection was followed by GO enrichment analysis of the clusters.

With a threshold for *p-value*≤0.001, the **best annotation strategy** detected clusters most similar to the ones reported in the previous analysis [12] (see Additional file 1: Table S3 for cluster comparison and https://biit.cs.ut.ee/funcexplorer/link/35872 for full details).

#### Yeast dataset

We conducted a similar comparison for the Yeast dataset analysed by Jin et al.[43]. Jin et al. analysed the transcriptome profiles of yeast exposed to transition metals using the K-means clustering with *K*=6 and functional enrichment analysis.

We found that the **F1 strategy** reveals more specific clusters (31 clusters that cover 28.96% of genes) with similar functions and expression profiles as reported previously [43] (see https://biit.cs.ut.ee/funcexplorer/link/cdeac for details). Because the 6 clusters from [43] had easily distinguishable expression profiles, we used the eigengene profiles to find the corresponding clusters from the funcExplorer results. The comparison is available in the Additional file 1: Table S4. In addition to gene clustering, the sample clustering in funcExplorer was successful in grouping the metals.

The goal of funcExplorer is to let the data speak for itself without imposing any assumptions. As there is no parameter in funcExplorer that allows to fix the number of clusters prior to analysis, detecting comparable set of 6 clusters is a challenge. However, setting a limit on the minimum cluster size similar to the ones reported by Jin et al. helps to get very similar results (e.g. the **best annotation strategy** with minimum cluster size 30 detects 8 clusters; https://biit.cs.ut.ee/funcexplorer/link/1a2c7).

To conclude, all the presented clustering results were obtained automatically. Without having domain specific knowledge we obtained similar biological results by simply uploading an expression dataset to funcExplorer. All the presented results are readily available for interpretation and further exploration at https://biit.cs.ut.ee/funcexplorer/user/83dfcffcba4067a43dc53025dbb1620a.

## Discussion

In this paper we have presented funcExplorer, a completely new tool that improves upon the idea of functional cluster detection first introduced in VisHiC[24]. We have introduced two novel approaches to extract functionally relevant clusters from a dendrogram. To demonstrate the utility of funcExplorer, we have reanalysed previously published datasets that are characterised by clustering and functional annotations. The showcases indicate that funcExplorer is able to automatically perform the initial manually conducted analysis of large-scale gene activity data, and uncover additional information. In addition, we analysed RNA-seq data from the GTEx project and detected tissue-specific clusters.

The tool is likely to be useful in the process of stating scientific hypotheses about the mechanisms that drive the co-expressed genes. Furthermore, by providing a quick functional overview for a selection of preprocessed datasets from ArrayExpress, funcExplorer is also beneficial when selecting a proper dataset for a specific question from the wide range of available datasets collected in public archives to be reanalysed. The utility of existing data collections is thereby magnified.

However, one of the key limitations of funcExplorer is that it does not provide an unambiguous solution for gene clustering. We have proposed three different strategies, but which approach is best remains unclear. This unclarity is largely due to the lack of a gold standard for cluster assessments in gene clustering.

Also, comparing novel gene clusters with previously published ones would require a robust and standardised approach that takes into account both the structure of clusters and annotations for fair assessment. In this paper we have used the Rand index and performed pairwise annotation comparison by hand. Both of these approaches have their shortcomings and further research is needed to come up with more automatic approach. A method that takes the relationships between annotations and clusters into account and can operate without the domain knowledge would be ideal.

To our knowledge, funcExplorer is the only functioning publicly available web application that combines hierarchical clustering with functional enrichment analysis and yields an informative visualisation for browsing at both a global and local level. The comparisons and case studies showed that funcExplorer is successful at emphasising the biologically relevant functional aspects of a dataset which are in accordance with previously conducted analyses. We believe that funcExplorer is capable of refining existing biological knowledge and revealing novel connections in a data-driven manner.

## Conclusions

In order to provide an initial comprehensive overview of the functional essence of high-throughput genomic data, funcExplorer carries out automated clustering and functional enrichment analysis. The visually compact result highlights the most relevant parts of the dataset at hand. In addition, the user can easily explore their experiment in more detail by using the interactive features provided by our tool. Furthermore, it is our hope that funcExplorer will advance scientific reproducibility by providing online results that can easily be shared among peers.

## Availability and Requirements

Project name: funcExplorerProject home page: https://biit.cs.ut.ee/funcexplorerOperating system: Platform independentOther requirements: Any browser with HTML5 supportProgramming language: PythonLicense: GNU GPL3Any restrictions to use by non-academics: None

## Additional files

Additional file 1 **Figure S1**. Features of funcExplorer and other similar tools. **Figure S2.** Theoretical maximum of -log10(*p*-value) score. The maximum enrichment score is limited by the cluster size due to the properties of hypergeometric distribution. The peak is achieved if the cluster size is equivalent to the size of the functional class. Similar behavior remains after multiple testing correction. Calculated for *N*=17,105. **Table S1.** Fixed-cut clusters of CLEANsmall. The number of clusters of size 5 to 1000 genes obtained after cutting at given distance (*#clusters*). The number of significantly enriched clusters is shown in the *#annot. clusters* column. **Table S2.** The clusters and corresponding marker genes as reported by Schmidt et al. [12]. **Table S3.** Comparison of funcExplorer results of Humoral dataset [12]. **Table S4.** Comparison of funcExplorer results of Yeast dataset [43]. (PDF 640 kb)

Additional file 2 Supplementary methods. Detailed descriptions of funcExplorer data preparation and calculations. (PDF 219 kb)

Additional file 3 List of significantly enriched GO functions for the cluster shown in Fig. 7. (XLSX 17 kb)

## Abbreviations

API: Application programming interface
GO: Gene ontology
GEO: Gene expression omnibus
GUI: Graphical user interface
HPA: Human protein atlas
HPO: Human phenotype ontology
KEGG: Kyoto encyclopedia of genes and genomes
SOM: Self-organizing map
TPM: Transcripts per million
