# Supplementary material for: funcExplorer: a tool for fast data-driven functional characterisation of high-throughput expression data
Source: BMC Genomics. 2018 Nov 14;19:817. doi: 10.1186/s12864-018-5176-x (PMC6236982; doi:10.1186/s12864-018-5176-x)
Supplement: Supplementary file 1 — Figure S1. Features of funcExplorer and other similar tools. Figure S2. Theoretical maximum of -log10(p-value) score. The maximum enrichment score is limited by the cluster size due to the properties of hypergeometric distribution. The peak is achieved if the cluster size is equivalent to the size of the functional class. Similar behavior remains after multiple testing correction. Calculated for N=17,105. Table S1. Fixed-cut clusters of CLEANsmall. The number of clusters of size 5 to 1000 genes obtained after cutting at given distance (#clusters). The number of significantly enriched clusters is shown in the #annot. clusters column. Table S2. The clusters and corresponding marker genes as reported by Schmidt et al. [12]. Table S3. Comparison of funcExplorer results of Humoral dataset [12]. Table S4. Comparison of funcExplorer results of Yeast dataset [43]. (PDF 640 kb) [file 12864_2018_5176_MOESM1_ESM.pdf]

Additional file 1 - Supplementary methods

Data preprocessing

After funcExplorer has converted the input data into the shape of an expression matrix, we exclude the rows with zero-variance across samples as they introduce unnecessary noise to the clustering. For missing values we offer a selection of solutions like replacing the missing values with user specified fixed value, mean value of the corresponding row or by applying K Nearest Neighbours (KNN) imputing from Python package *fancyimpute* [1] using 5 nearest rows. Rows with missing values are excluded if none of the methods above is selected.

funcExplorer does not explicitly handle the normalisation of input data as normalising the raw data is highly dependent on the peculiarity of the data. We assume that the raw data has already passed some preparation steps like adjustment for the background, normalisation and summarisation which results in expression values in the matrix cells. For example, in case of microarray data, we recommend to use the approach that was used for our public datasets (described in previous section). A comprehensive overview of existing approaches is written by Gentleman *et al.* [2]. However, subsequent data transformations, such as centering and standardising, are provided as a user selectable input in funcExplorer. In order to improve the readability of the heatmap and keep the clustering stable, we recommend to standardise the gene expression matrix to have zero-mean and unit variance across the samples before hierarchical clustering is performed. Selection of other data transformations like median standardisation and centering are also available.

We handle gene/protein activity data from RNA-seq, microarray, ProtoArray or any experiments that can be brought into the form of a data matrix where the identifiers in the rows are recognisable in g:Profiler. This includes majority of gene identifiers provided by Ensembl, Uniprot, RefSeq, Entrez, Illumina, Affymetrix, etc. At least 13 types of IDs are supported for all of the 213 species available in g:Profiler, and at least 40 types of IDs for more than 50 species.

The RNA-seq quantification is based on discrete read counts rather than continuous measures of expression levels. funcExplorer includes methods such as variance-stabilising transformation and normalisation for sequencing depth, from Bioconductor package DESeq2 [3] for normalisation and transformation of uploaded raw read counts so that we could apply the same methods as in case of microarray data. A good overview of the topic and other relevant methods has been written by Datta *et al.* [4]. Therefore, in case of RNA-seq data, funcExplorer accepts either raw read count matrix or already preprocessed data as input.

Multiple testing

Since the enrichment tests are performed for all annotation sets available in the g:Profiler database (e.g. more than 30,000 annotation terms can be considered for analysis of human gene clusters), multiple testing correction is applied in order to reduce the amount of false positives resulting from numerous enrichment tests. The special correction that takes into account the set structure of annotations is used by default (defined as g:SCS method in g:Profiler [5]); nevertheless, users can also choose to apply standard methods like Bonferroni correction or Benjamini–Hochberg False Discovery Rate [6]. A function is considered to show significant over-representation if $p-value \leq 0.05$ after multiple comparison correction. All functional terms that remain statistically significant after multiple testing correction are incorporated in the computation of the enrichment score in funcExplorer.

Choosing the background set

Dependent on the data and question at hand, it is important to choose a suitable background set for testing. In g:Profiler and therefore also in funcExplorer, by default, the background is a set of all known annotated genes in the respective genome. However, such approach may give misleading results when the user is initially focusing on a narrower set of genes. Choosing the set of genes in the uploaded dataset as a background in the hypergeometric tests instead of the whole genome can in several occasions result in more accurate statistics and specific annotations. For example, if the number of genes and corresponding probesets of a platform is considerably smaller than the number of known genes. The choice of suitable background set is dependent on the research question and can be selected by the user while uploading the data.

Calculating the enrichment scores

g:Profiler estimates the p-value p_T of a single process, pathway or other annotation T based on values shown in a contingency table 1. Given that there are k genes in a cluster of n genes with functional annotation T, and there are M genes annotated with T among the total of N genes in the genome (or the dataset at hand, depending on the user preference),

$$p_T = \sum_{i=k}^{\min(n,M)} \frac{\binom{M}{i}\binom{N-M}{n-i}}{\binom{N}{n}}. \quad (1)$$

Table 1: Contingency table of enrichment test. The values used to calculate the p-value in Eq. 1 of function T in a cluster $\mathbb{C}$.

	genes in $\mathbb{C}$	genes not in $\mathbb{C}$	Total
genes in T	k	M-k	M
genes not in T	n-k	N-n-M+k	N-M
Total	n	N-n	N

The **best annotation strategy** calculates the enrichment of a cluster based on a maximum of negative log p-value scores calculated over all significant annotation classes T_j of the cluster after multiple correction. In other words, each cluster $\mathbb{C}$, which is significantly enriched in j biological functions, is assigned an enrichment score

$$m_{\mathbb{C}} = \max_{T_j \in \mathbb{C}} \left(-\log_{10}(p_{T_j}) \right). \tag{2}$$

F1 score is the harmonic mean of the precision and recall with values ranging from 0 to 1 (perfect precision and recall) [7]. The **F1 strategy** uses the corresponding F1 score of cluster $\mathbb{C}$ with respect to function T_j calculated with the equation:

$$F1(\mathbb{C}, T_j) = 2 \cdot \frac{precision(\mathbb{C}, T_j) \cdot recall(\mathbb{C}, T_j)}{precision(\mathbb{C}, T_j) + recall(\mathbb{C}, T_j)} \tag{3}$$

Precision is the fraction of correct predictions out of the total number of predictions. In our case it is the ratio between the genes in a cluster $\mathbb{C}$ that are also annotated in a function T_j and the total number of genes in that cluster. Recall is the fraction of correct predictions from the total number of true positives. In our case it is the ratio between the number of genes in a cluster $\mathbb{C}$ that were also found annotated in a function T_j and the total number of genes annotated in that function. Therefore, the corresponding values for recall and precision with respect to cluster $\mathbb{C}$ and function T_j can be calculated from the values given in table 1:

$$recall(\mathbb{C}, T_j) = \frac{k}{M}; \qquad precision(\mathbb{C}, T_j) = \frac{k}{n} \tag{4}$$

Similar to the best annotation strategy, for every cluster $\mathbb{C}$ in a dendrogram we assign a score which is the maximum value of corresponding F1 scores over all significant functions T_j enriched in the cluster:

$$f_{\mathbb{C}} = \max_{T_j \in \mathbb{C}} \left(F1(\mathbb{C}, T_j) \right). \tag{5}$$

Enrichment-driven pruning algorithm

Two-stage greedy algorithm from [8] is applied in order to determine the clusters based on functional annotations.

(1) First we look for clusters with the strongest functional class signal using best annotation score $m_{\mathbb{C}}$ or F1 strategy score $f_{\mathbb{C}}$, or alternatively, selecting the first cluster in a branch that reveals any enrichment signal.

Greedily starting from the cluster that has the highest strategy score we call this cluster informative and continue our search in a decreasing order of the strategy score from neighboring branch. A cluster is not considered in the search if any of its child or parent clusters is already noted as an informative cluster. The identified clusters are shown in the final output as color-coded rectangles, where colors represent the functional categories.

(2) Next, we detect clusters that have poor or no functional enrichment. We traverse the dendrogram recursively starting from the root, compressing all clusters except the ones that contain informative cluster as child nodes. By default, poorly annotated clusters and corresponding expression profiles are hidden from the results. However, including these to the output is made optional for the users.

Public datasets

Robust Multi-array Average (RMA) normalisation [9] was performed on the Affymetrix raw data with the Bioconductor *affy* package [10] using the default parameters. Biological annotations of the datasets are also included as annotated according to the Minimum Information About a Microarray Experiment (MIAME) standard [11]. The data loading and preprocessing was performed for the MEM tool [12], and shared to funcExplorer. The expression matrices were standardised before funcExplorer analysis.

The CLEAN datasets

The CLEANsmall dataset was downloaded from http://eh3.uc.edu/clean. The details of preprocessing the data are described in the Methods section of article [13]. The dataset consists of 1,422 preselected genes and 808 samples.

The CLEANtotal datasets were obtained from the MEM tool preprocessed dataset collection with corresponding ArrayExpress accession numbers: E-GEOD-1456, E-GEOD-3494, E-GEOD-7390 and E-GEOD-11121. Therefore, the expression matrices were previously RMA normalised. We standardised the matrices and merged them into CLEANtotal dataset. Gene IDs were converted to ENSEMBL IDs and the genes with same identifiers were merged by average expression values. Rows with zero-variance were filtered out. The final datase used for this analysis contains 13,351 genes.

The GTEx dataset

The GTEx dataset was downloaded from https://www.gtexportal.org/home/datasets. The dataset consists of the median TPM values by tissue. The dimensions of the data matrix, after excluding rows with zero-variance, are 53 columns and 42,548 rows.

Before the analysis, the TPM values were transformed to $\log_2(TPM + 1)$ followed by centering and standardisation. With two-color palette these transformations help to highlight the low-and high-expressed genes in our setting. Samples were not clustered.

Humoral dataset

The Humoral dataset was also obtained from the MEM tool preprocessed dataset collection with accession number E-GEOD-11121. We applied median standardisation to the data which is similar to the data transformation applied by Schmidt *et al.* [14]. For the comparison analysis we used only the subset of 2,579 genes as was analysed by Schmidt *et al.* [14]. We did not cluster the samples.

Arabidopsis and Yeast dataset

The Arabidopsis and Yeast datasets were extracted from the supplementary material of the corresponding original studies, [15] and [16]. No data transformation was applied. The samples of Yeast data were reordered according to hierarchical clustering.

The Rand index

The Rand index [17] from R package *mcclust* [18] was used to compare the CLEAN and funcExplorer clusterings. Rand index is based on how often the two clusterings agree in the treatment of pairs of observations, genes in our case, where agreement means that two genes are in or are not in the same cluster in both of the clusterings. The adjusted Rand index adjusts for the expected number of chance agreements.

The Rand index has a value between 0 and 1, with 0 indicating that the two data clusterings do not agree on any pair of observations and 1 indicating that the data clusterings are identical. The range of values of adjusted Rand index is from -1 to 1, where -1 means that the agreement between two clusterings is less than what is expected from a random result and 1 means they are identical.

Technical details

funcExplorer is publicly available web service that is built on the Flask framework. An Apache HTTPS server with Python and JavaScript technologies are used for the funcExplorer website. The back end, including the enrichment-driven pruning algorithm, is implemented in Python 2.7 and runs on network docker containers. The intermediate data (user details, annotations, data description, etc) as well as web caches are stored in PostgreSQL databases. The tasks in funcExplorer are handled asynchronously by Celery workers. The source of Hybrid hierarchical clustering was obtained from http://carrier.gnf.org/publications/cluster/index.html. The main codebase, including Dockerfiles, all the back end functions and web services, is publicly available in https://gl.cs.ut.ee/biit/funcexplorer_public. For function enrichment analysis the g:Profiler is used through server-side local API, thus fully standalone version of funcExplorer can not be built yet. Currently there are still limitations on sharing services relying on big and partly licensed data and server-specific codebases.

The results of funcExplorer analysis can be explored in all modern web browsers. The GUI of funcExplorer uses predominantly D3.js [19] as its visualisation component in client side. It handles most of the rendering of the data within the web browser using a combination of standard HTML “div” and “canvas” elements. The data are sent to the web browser as JSON objects.

References

[1] Rubinsteyn, A., Feldman, S.: fancyimpute: Version 0.0.16. (2016). doi:10.5281/zenodo.51773

[2] Gentleman, R., Carey, V., Huber, W., Irizarry, R., Dudoit, S.: Bioinformatics and computational biology solutions using R and Bioconductor. Springer (2006)

[3] Love, M.I., Huber, W., Anders, S.: Moderated estimation of fold change and dispersion for RNA-seq data with DESeq2. Genome biology **15**(12), 550 (2014)

[4] Datta, S., Nettleton, D.: Statistical analysis of next generation sequencing data. Springer (2014)

[5] Reimand, J., Arak, T., Adler, P., Kolberg, L., Reisberg, S., Peterson, H., Vilo, J.: g: Profiler—a web server for functional interpretation of gene lists (2016 update). Nucleic acids research **44**(W1), 83–89 (2016)

[6] Benjamini, Y., Yekutieli, D.: The control of the false discovery rate in multiple testing under dependency. Annals of statistics, 1165–1188 (2001)

[7] van Rijsbergen, C.J.: Information Retireval (1979)

[8] Krushevskaya, D., Peterson, H., Reimand, J., Kull, M., Vilo, J.: VisHiC—hierarchical functional enrichment analysis of microarray data. Nucleic acids research **37**(suppl_2), 587–592 (2009)

[9] Irizarry, R.A., Hobbs, B., Collin, F., Beazer-Barclay, Y.D., Antonellis, K.J., Scherf, U., Speed, T.P.: Exploration, normalization, and summaries of high density oligonucleotide array probe level data. Biostatistics **4**(2), 249–264 (2003)

[10] Gautier, L., Cope, L., Bolstad, B.M., Irizarry, R.A.: affy—analysis of Affymetrix GeneChip data at the probe level. Bioinformatics **20**(3), 307–315 (2004)

[11] Brazma, A., Hingamp, P., Quackenbush, J., Sherlock, G., Spellman, P., Stoeckert, C., Aach, J., Ansorge, W., Ball, C.A., Causton, H.C., *et al.*: Minimum information about a microarray experiment (MIAME)—toward standards for microarray data. Nature genetics **29**(4), 365–371 (2001)

[12] Adler, P., Kolde, R., Kull, M., Tkachenko, A., Peterson, H., Reimand, J., Vilo, J.: Mining for coexpression across hundreds of datasets using novel rank aggregation and visualization methods. Genome biology **10**(12), 139 (2009)

[13] Freudenberg, J.M., Joshi, V.K., Hu, Z., Medvedovic, M.: Clean: Clustering enrichment analysis. BMC bioinformatics **10**(1), 234 (2009)

[14] Schmidt, M., Böhm, D., von Törne, C., Steiner, E., Puhl, A., Pilch, H., Lehr, H.-A., Hengstler, J.G., Kölbl, H., Gehrmann, M.: The humoral immune system has a key prognostic impact in node-negative breast cancer. Cancer research **68**(13), 5405–5413 (2008)

[15] Chupeau, M.-C., Granier, F., Pichon, O., Renou, J.-P., Gaudin, V., Chupeau, Y.: Characterization of the early events leading to totipotency in an arabidopsis protoplast liquid culture by temporal transcript profiling. The Plant Cell, 113 (2013)

[16] Jin, Y.H., Dunlap, P.E., McBride, S.J., Al-Refai, H., Bushel, P.R., Freedman, J.H.: Global transcriptome and deletome profiles of yeast exposed to transition metals. PLoS genetics **4**(4), 1000053 (2008)

[17] Hubert, L., Arabie, P.: Comparing partitions. Journal of classification **2**(1), 193–218 (1985)

[18] Fritsch, A.: mcclust: Process an MCMC Sample of Clusterings (2009). http://cran.r-project.org/package=mcclust

[19] Bostock, M., Ogievetsky, V., Heer, J.: D^3 data-driven documents. IEEE transactions on visualization and computer graphics **17**(12), 2301–2309 (2011)
