# Supplementary material for: funcExplorer: a tool for fast data-driven functional characterisation of high-throughput expression data
Source: BMC Genomics. 2018 Nov 14;19:817. doi: 10.1186/s12864-018-5176-x (PMC6236982; doi:10.1186/s12864-018-5176-x)
Supplement: Supplementary file 2 — Supplementary methods. Detailed descriptions of funcExplorer data preparation and calculations. (PDF 219 kb) [file 12864_2018_5176_MOESM2_ESM.pdf]

Additional file 2 - Supplementary tables and figures

	funcExplorer	Expander	AMEN	CLEAN	HCE	Clustergrammer	ClusterProfiler
**Provides clustering**	Yes	Yes	Yes	Yes	Yes	Yes	No
**Provides enrichment analysis**	Yes	Yes	Yes	Yes	Yes	No, outsources Enrichr one domain and cluster at a time	Yes
**Functional domains**	GO, KEGG, REAC, TF, miRNA, CORUM, HPA, HPO	GO	GO	GO, KEGG, Transfac	GO	GO, HPO, KEGG, Reactome, PANTHER, CORUM and many more	KEGG, GO
**Online/desktop/R**	Online	Desktop	Desktop	R package	Desktop	Online	R package
**Provides visualisation**	Yes	Yes	Yes	No, outsources FTreeView clustering browser	Yes	Yes	Yes
**Interactive**	Yes	Yes	Yes	Yes*, FTreeView is interactive	Yes	Yes	No
**Data preprocessing**	Yes	Yes	Yes	No	Yes	No	No
**Functioning**	Yes	Yes	No	Yes	Yes	Yes	Yes
**Needs additional downloads**	No	Yes	Yes	Yes	Yes	No	No

Figure S1: **Features of funcExplorer and other similar tools.**

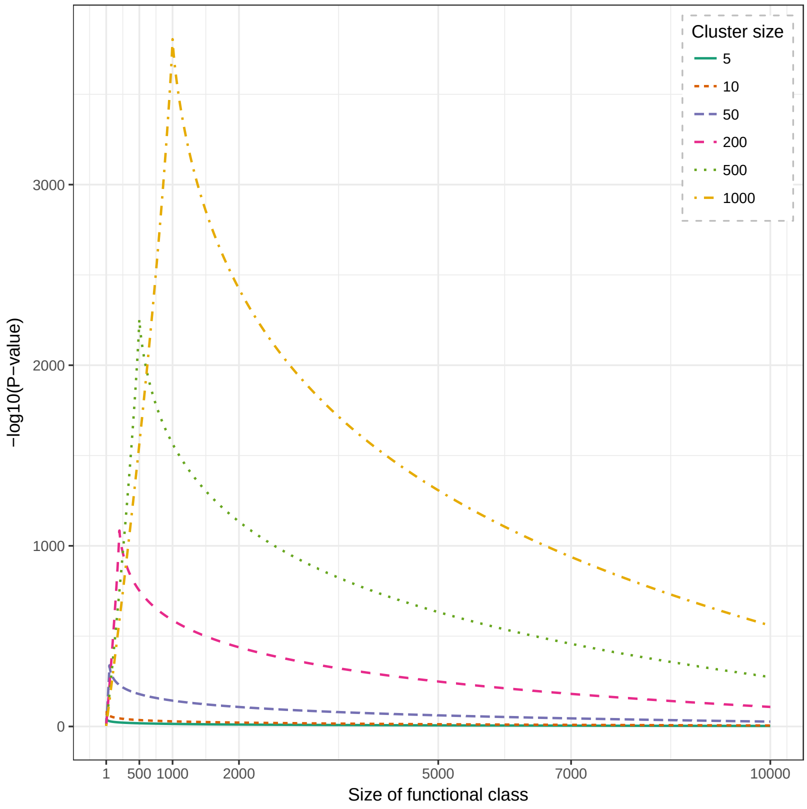

Figure S2: **Theoretical maximum of -log10(p-value) score.** The maximum enrichment score is limited by the cluster size due to the properties of hypergeometric distribution. The peak is achieved if the cluster size is equivalent to the size of the functional class. Similar behavior remains after multiple testing correction. Calculated for $N = 17,105$.

Table S1: **Fixed-cut clusters of CLEANsmall.** The number of clusters of size 5 to 1000 genes obtained after cutting at given distance (*#clusters*). The number of significantly enriched clusters is shown in the *#annot. clusters* column.

i	distance	#clusters	#annot. clusters
1	0.3	2	0
2	0.35	4	4
3	0.4	6	4
4	0.45	6	5
5	0.5	9	9
6	0.55	13	10
7	0.6	13	12
8	0.65	14	12
9	0.7	18	14
10	0.75	21	15
11	0.8	21	16
12	0.85	28	18
13	0.9	33	24
14	0.95	35	23
15	1	35	22
16	1.05	34	23
17	1.1	32	20
18	1.15	35	22
19	1.2	36	26
20	1.25	36	26
21	1.3	33	28
22	1.35	33	27
23	1.4	30	21
24	1.45	32	21
25	1.5	27	18
26	1.55	25	18
27	1.6	21	16
28	1.65	20	16
29	1.7	18	16
30	1.75	12	11
31	1.8	11	10
32	1.85	9	8
33	1.9	2	1
34	1.95	1	1
35	2	0	0

Table S2: The clusters and corresponding marker genes as reported by Schmidt *et al.* [1]

Class	Marker genes
Basal	KRT5, KRT17
Cellular Immunsystem	TRA@, TRB@, TRD@, CD8A
Humoral Immunsystem	IGHM, IGHG, IGKC
Interferon	IFI27, IFI35, IFIT1
Proliferation	CCNB2, UBE2C, STK6, MKI67
Estrogen receptor	ESR1, MAPT, PGR, SCUBE2
Chr 17	ERBB2, GRB7
Stromal	COL1A1, COL5A1, COL6A1
Normal like	FABP4, PPARG
Jun - Fos	JUN, FOS
Transcription	None reported

Table S3: **Comparison of funcExplorer results of Humoral dataset [1]**

**Schmidt *et al.* clusters**		**Corresponding funcExplorer clusters**	
**Cluster name**	**Reported functions**	**Cluster ID (size)**	**Top GO terms BP, CC, MF**
1. Basal-like (228 genes)	Intermediate filament, **epidermis development**, structural constituent of cytoskeleton, **morphogenesis**, **central nervous system development**, zinc ion binding	ID: 156 (290 genes)	Tissue development, -, -
2. T-cell (134 genes)	**Immune response**, **cellular defense response**, **inflammatory response**, **chemotaxis**, MHC protein binding, **intracellular signaling cascade**, **T-cell activation**	ID: 459 (297 genes)	Immune system process, side of membrane, cytokine receptor activity
3. B-cell (60 genes)	**Antigen binding**, **immune response**, **membrane fraction**		
4. Interferon (36 genes)	**Immune response**, **response to virus**, nucleotidyltransferase activity, RNA binding, cytoplasm	ID: 871 (45 genes)	Response to virus, -, -
5. Proliferation (97 genes)	**Mitosis**, cytokinesis, nucleus, **cell cycle**	ID: 1842 (32 genes)<br>ID: 1213 (44 genes)<br>ID: 2143 (20 genes)	Cell cycle, spindle, -<br>Mitotic cell cycle process, chromosome, -<br>Mitotic nuclear division, spindle, microtubule motor activity
6. ER (luminal) (121 genes)	DNA binding	ID: 27 (551 genes)	Positive regulation of extrinsic apoptotic signaling pathway, extracellular exosome, protein dimerization activity
7. Chr 17 (ERBB2) (58 genes)	-	-	-
8. Stromal (175 genes)	**Extracellular matrix structural constituent**, **extracellular matrix**, phosphate transport, **collagen**, cell adhesion	ID: 1029 (198 genes)	Extracellular matrix organization, extracellular matrix, extracellular matrix structural constituent
9. Normal-like (285 genes)	Muscle development, insulin-like growth factor receptor binding, trans-1,2-dihydrobenzene-1,2-diol dehydrogenase activity, neurogenesis	ID: 1764 (29 genes)	Lipid metabolic process, lipid droplet, -
10. Jun-Fos (58 genes)	Hemoglobin complex, oxygen transporter activity, oxygen transport, heme binding, oxygen binding, transcription factor activity	-	-
11. Transcription (129 genes)	Nuclear mRNA splicing via spliceosome, RNA binding, nucleic acid binding, RNA processing	-	-

* Note: The **best annotation strategy** with *p-value* ≤ 0.001 and no upper limit for term size; cluster IDs ordered by the enrichment score; cluster sizes are given in the brackets; the characteristic functions that also appear significant in the corresponding funcExplorer clusters are highlighted in **bold**; Top functions are from GO biological process (BP), cellular component (CC) and molecular function (MF)

Table S4: **Comparison of funcExplorer results of Yeast dataset [2]**

**Jin *et al.* clusters**		**Corresponding funcExplorer clusters**	
**Cluster ID (size)**	**Top functions from g:Profiler**	**Cluster ID (size)**	**Top GO terms BP, CC, MF**
I (388 genes)	Transmembrane transport, ion transport, iron ion homeostasis, cell periphery, plasma membrane, vacuole, cell periphery, sequence-specific DNA binding, transporter activity	ID: 400 (10 genes)	iron chelate transport, integral component of plasma membrane, water channel activity
		ID: 348 (6 genes)	regulation of establishment or maintenance of cell polarity, -, -
		ID: 174 (19 genes)	arsenate ion transmembrane transport, oxidoreductase complex, -
		ID: 147 (11 genes)	steroid metabolic process, -, -
		ID: 1213 (12 genes)	cytoplasmic translation, cytosolic ribosome, structural constituent of ribosome
II (39 genes)	Sulfur compound metabolic process, celluar amino acid metabolic process, sulfite reductase complex (NADPH), oxidoreductase activity	ID: 1147 (5 genes)	Sulfate assimilation, sulfite reductase complex (NADPH), -
		ID: 1245 (5 genes)	disaccharide catabolic process, -, oligo-1,6-glucosidase activity
		ID: 1193 (13 genes)	sulfur compound transport, -, sulfur compound transmembrane transporter activity
		ID: 1035 (33 genes)	response to chemical, -, -
III (175 genes)	Carbohydrate metabolic process, small molecule metabolic process, generation of precursor metabolites and energy, mitochondrion, oxidoreductase complex	ID: 1116 (11 genes)	tricarboxylic acid cycle, mitochondrial respiratory chain, -
		ID: 1260 (5 genes)	-, -, oxidoreductase activity, acting on CH-OH group of donors
IV (181 genes)	Nucleolus, preribosome, ribosome biogenesis, rRNA metabolic process, snoRNA binding	ID: 1266 (5 genes)	rRNA processing, Rix1 complex, -
		ID: 1268 (5 genes)	maturation of SSU-rRNA from tricistronic rRNA transcript (SSU-rRNA, 5.8S rRNA, LSU-rRNA), 90S preribosome, snoRNA binding
		ID: 1185 (5 genes)	ribosomal large subunit biogenesis, preribosome, -
V (447 genes)	Nuclear nucleosome, nucleosome, DNA packaging complex	ID: 248 (6 genes)	monosaccharide transmembrane transport, -, alpha-glucoside:proton symporter activity
		ID: 428 (6 genes)	reactive oxygen species metabolic process, -, glyceraldehyde-3-phosphate dehydrogenase (NAD+) (phosphorylating) activity
		ID: 46 (5 genes)	-, -, acid phosphatase activity
		ID: 521 (11 genes)	nucleosome assembly, nuclear nucleosome, nucleosomal DNA binding
		ID: 434 (14 genes)	protein localization by the Cvt pathway, -, -
VI (110 genes)	Oxidoreductase activity, response to oxidative stress, response to chemical, catalytic activity, response to stress, protein folding	ID: 581 (7 genes)	-, -, oxidoreductase activity acting on NAD(P)H, quinone or similar compound as acceptor
		ID: 480 (21 genes)	response to heat, -, -

* Note: The **F1 strategy** with *p-value* ≤ 0.01 and no upper limit for term size; cluster IDs ordered by the enrichment score; cluster sizes are given in the brackets; Top functions are from GO biological process (BP), cellular component (CC) and molecular function (MF); g:Profiler was used to annotate the initial clusters due to inconsistent reporting in the original study [2]

References

[1] Schmidt, M.,*et. al*: *The humoral immune system has a key prognostic impact in node-negative breast cancer*. Cancer research **68**(13), 5405–5413 (2008)

[2] Jin, Y.H.,*et. al*: *Global transcriptome and deletome profiles of yeast exposed to transition metals*. PLoS genetics **4**(4), 1000053 (2008)
